# Supplementary material for: Sexual rejection via a vomeronasal receptor-triggered limbic circuit
Source: Nat Commun. 2018 Oct 26;9:4463. doi: 10.1038/s41467-018-07003-5 (PMC6203846; doi:10.1038/s41467-018-07003-5)
Supplement: Supplementary file 1 — Supplementary Information [file 41467_2018_7003_MOESM1_ESM.pdf]

Supplementary Information

Sexual rejection via a vomeronasal receptor-triggered limbic circuit

Osakada et al.

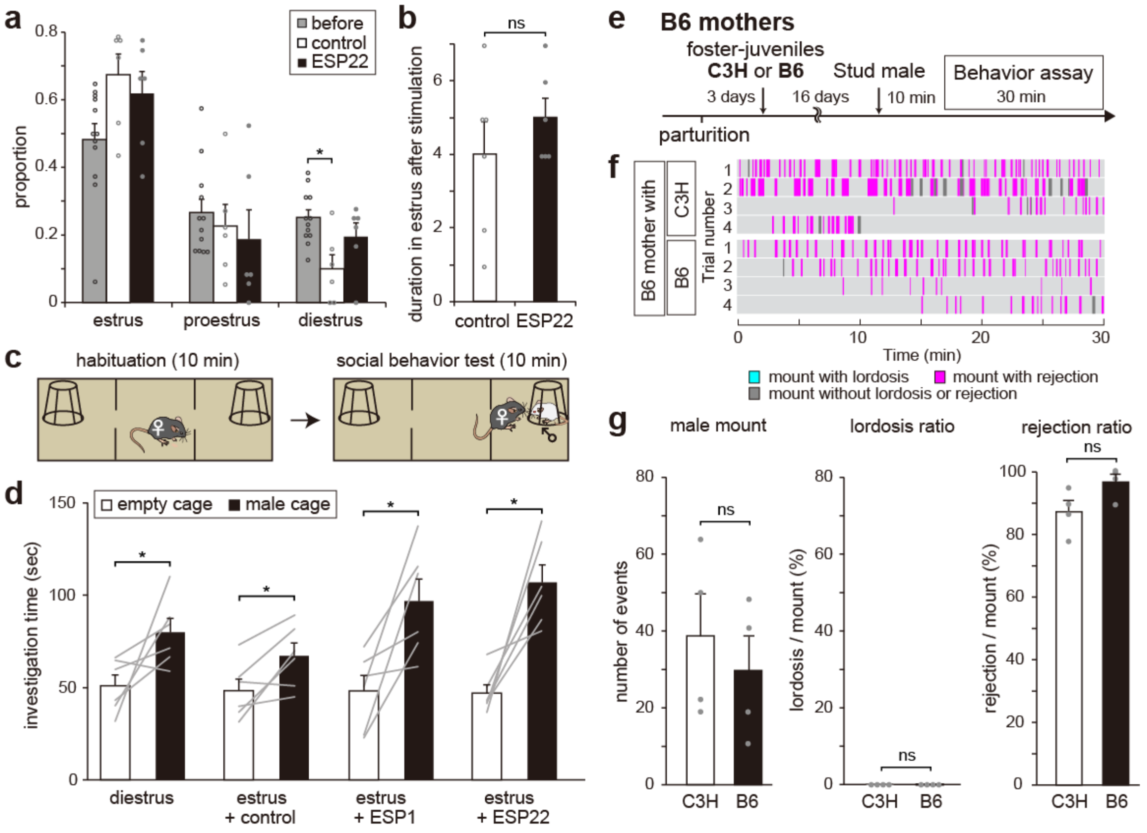

Supplementary Fig. 1 Effect of ESP22 on C57BL/6 virgin female mice and lactating mothers. **a** Vaginal smears of adult female mice were assessed daily, and ESP22 or control buffer was added to their drinking water for over 10 days (as described in **Fig. 2d**). We quantified the proportion of female mice in estrus, proestrus, and diestrus phase in three groups prior to supplying control buffer or ESP22 (before) and after supplying control buffer (control) or ESP22 (ESP22). No major differences were observed, which suggests that ESP22 does not affect the estrus cycle of adult female mice. Error bars, S.E.M. before, n = 12; control, n = 6; and ESP22, n = 6. *$p<0.05$ by Steel-Dwass test. **b** Duration in estrus phase after supplying control buffer or ESP22 in the drinking water. In both conditions, female mice spent 4–5 days in the estrus phase. Error bars, S.E.M. n = 6 animals each for control buffer and ESP22 groups. ns, not significant. Statistical analysis by Wilcoxon rank sum test. **c** Schematic illustration of three-chamber social behavior assay. An empty cage was placed in both the left and right rooms. This assay used adult female mice in diestrus and estrus that were pre-exposed to control buffer, ESP1, or ESP22. After the female mouse was allowed 10 min of habituation in the test chamber, a strange ICR male mouse was randomly introduced into one of two empty cages. The social investigation time spent by the female mouse was counted over a 10-min period. **d** Quantification of the investigation time of the empty and male cages. Gray lines represent individual data. ESP22 pre-exposed female mice did not exhibit any impaired social motivation regarding the investigation of the strange male mouse.

Instead, these mice tended to exhibit enhanced investigation time of the male cage versus the empty cage, a trend that was also observed for the female mice that were pre-exposed to ESP1. Error bars, S.E.M. *$p<0.05$ by Wilcoxon signed rank test. **e** Timeline for sexual behavior assays using C57BL/6 lactating mothers with C3H/HeJ (C3H, ESP22-) or C57BL/6J (B6, ESP22+) juvenile mice. **f, g** Raster plots and quantification are the same as detailed in **Fig. 1b, c**. n = 4. Error bars, S.E.M. ns, not significant. Statistical analysis by Wilcoxon rank sum test with Holm correction.

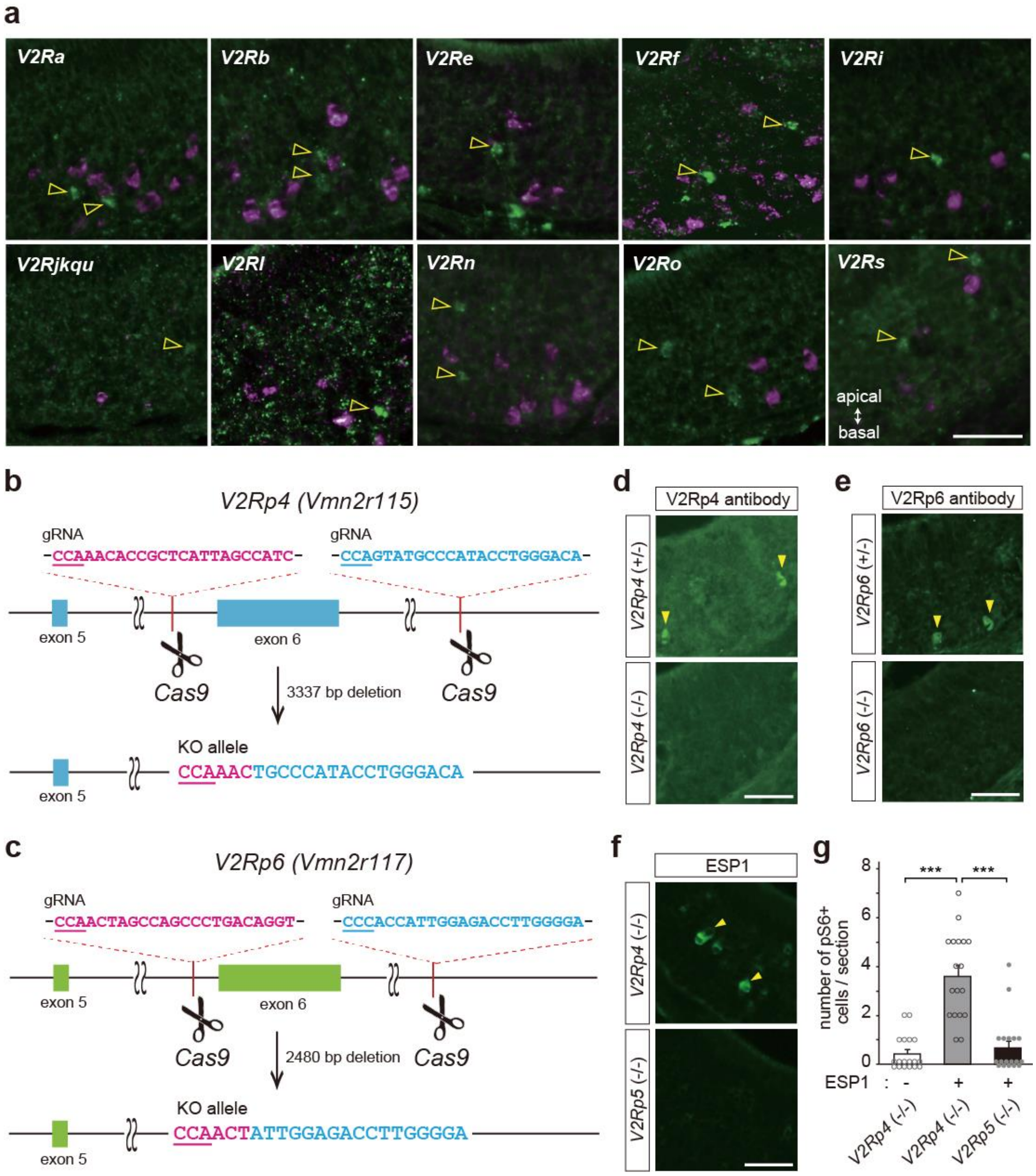

Supplementary Fig. 2 Screening of the candidate V2Rs for ESP22. **a** Representative images of a VNO section from an ESP22-stimulated mouse labeled by ISH with *Egr1* (green) and *V2R* clade-specific cRNA probes (magenta). Open arrowheads show the singly-labeled *Egr1*-positive cells. Scale bar, 50 µm. **b, c** Schematic diagram illustrating the generation of *V2Rp4* (a.k.a. *Vmn2r115*) or *V2Rp6* (a.k.a. *Vmn2r117*) deficient mice. Two guide RNAs (shown in magenta and cyan) were designed to induce the Cas9-mediated double strand breaks flanking exon 6, which encodes the entire transmembrane domain of V2R. The non-homologous end joining following the dual double strand breaks would result in the deletion of the entire exon 6. Junction sequence was confirmed by genomic PCR. **d, e** V2Rp4 or V2Rp6 immunostaining of VNO sections from

each mutant mouse. Arrowheads denote the expression of V2Rp4 or V2Rp6. Scale bar, 50 μm. In the homozygous mutant mice, corresponding V2R expression completely disappeared. Scale bar, 50 μm. **f** Representative immunohistochemical images of pS6-expressing VSNs in ESP1-stimulated *V2Rp4*$^{-/-}$ and *V2Rp5*$^{-/-}$ mice. Arrowheads denote representative pS6-positive VSNs. Scale bar, 50 μm. **g** The number of pS6-positive cells per VNO section. For each genotype, 18 sections from each of three animals were quantified. Error bars, S.E.M. ***$p<0.001$ by Steel-Dwass test.

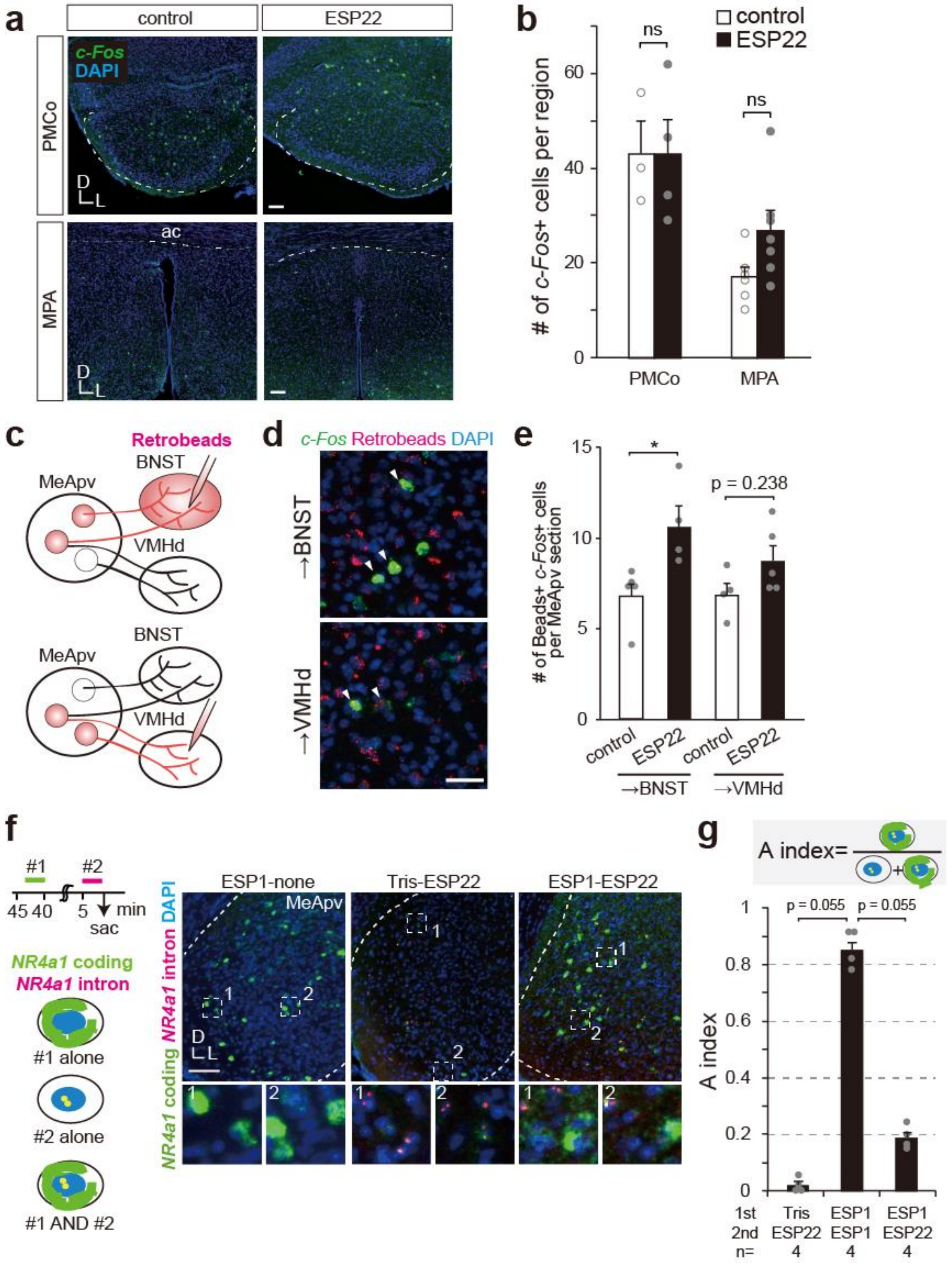

Supplementary Fig. 3 The patterns of pheromone-dependent activation in higher brain regions were typically different between ESP22 and ESP1. **a** Representative ISH sections of PMCo and MPA from female mice stimulated with control buffer or ESP22. *c-Fos* cRNA probe (green) was used in conjunction with nuclear DAPI staining (blue). Scale bar, 100 µm. **b** Quantification of *c-Fos*-expressing neurons in the PMCo and MPA. The number of sections counted in each animal

was: PMCo, 8; and MPA, 5 (containing left and right hemispheres). Error bars, S.E.M. *n* = 3–7. ns, not significant. Statistical analysis by Wilcoxon rank sum test. **c** Schematic illustration of the experimental setup for projection-specific labeling. **d** Representative MeApv coronal sections showing *c-Fos* expression in neurons projecting to BNST (→BNST) and VMHd (→VMHd) of female mice following ESP22 exposure. White arrowheads indicate Retrobeads-positive cells expressing *c-Fos*. **e** Number of *c-Fos*- and Retrobeads-positive cells per MeApv section in female mice. *n* = 4–5. *$p<0.05$ by Wilcoxon rank sum test. **f** Schematic illustration of catFISH analysis and representative coronal sections from catFISH analysis in the MeApv. The stimulation conditions are shown in each image (e.g., "ESP1-none" corresponds to stimulation with ESP1 followed by no stimulation). Insets show high-magnification images of the two boxed areas in each panel. D, dorsal, L, lateral. Blue represents DAPI nuclear staining. Scale bar, 100 μm. **g** Proportion of dual-labeled cells among nuclear *NR4a1*-positive cells (A-index). Error bars, S.E.M. *n* = 4. Statistical analysis by Steel-Dwass test.

a

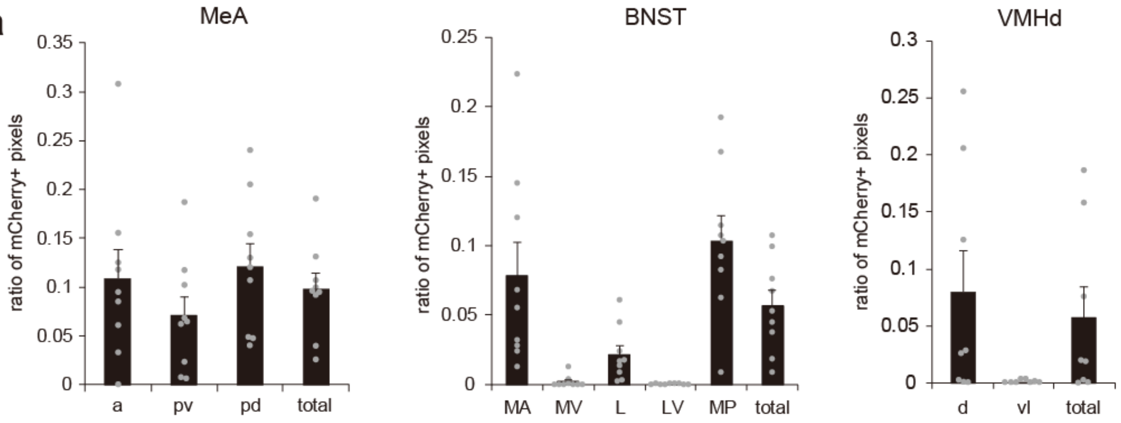

b

	MeA				BNST						VMH		
	a	pv	pd	total	MA	MV	L	LV	MP	total	d	vl	total
R	-0.5813	-0.668	-0.0739	-0.6312	-0.5111	-0.1395	-0.5523	-0.3675	-0.6229	-0.6659	0.3974	0.4348	0.4505
p	0.0779	0.0347	0.839	0.0503	0.1311	0.7006	0.0977	0.2961	0.0544	0.0355	0.2894	0.2421	0.2235

Supplementary Fig. 4 Additional analysis of loss-of-function experiments. **a** Proportion of mCherry-positive pixels in each sub-region and whole MeA, BNST, and VMHd that were targeted in loss-of-function experiments. Error bars, S.E.M. As shown, mCherry (hM4Di) was broadly targeted to MeAa, MeApv and MeApd. In the BNST, mCherry was mainly targeted to MA, L, and MP subregions with minimum expression in MV or LV subregions. In the VMH, as SF1-Cre was used to restrict transgene expression only in the VMHd, almost all mCherry positive neurons were located in the VMHd. Each experimental animal showed certain variability in the targeting efficiency in each subregion, allowing us to analyze correlation coefficient (R) between ratio of mCherry+ pixel (inferring hM4Di targeting efficiency) and Δrejection ratio (showing the effect of ESP22). Some of these analyses were graphically shown in Figures 5e, 5h, and 5k. **b** Table in this panel shows R values for each subregion and total MeA, BNST and VMH, with p-values (p) under the null-hypothesis that there is no correlation ($R = 0$). Red text represents $p<0.05$. In the MeA, hM4Di-targeting efficiency in MeApv, but not MeApd, tended to negatively correlate with Δrejection ratio, suggesting that loss-of-function of MeApv impaired ESP22-induced rejection. In the BNST, MA, L, and MP all showed trend of negative correlation, but R-value was only significantly different from zero when entire BNST data were pooled (total). In VMH, none of subregion showed negative correlation. Of note, we did not correct multiple comparisons in this table.

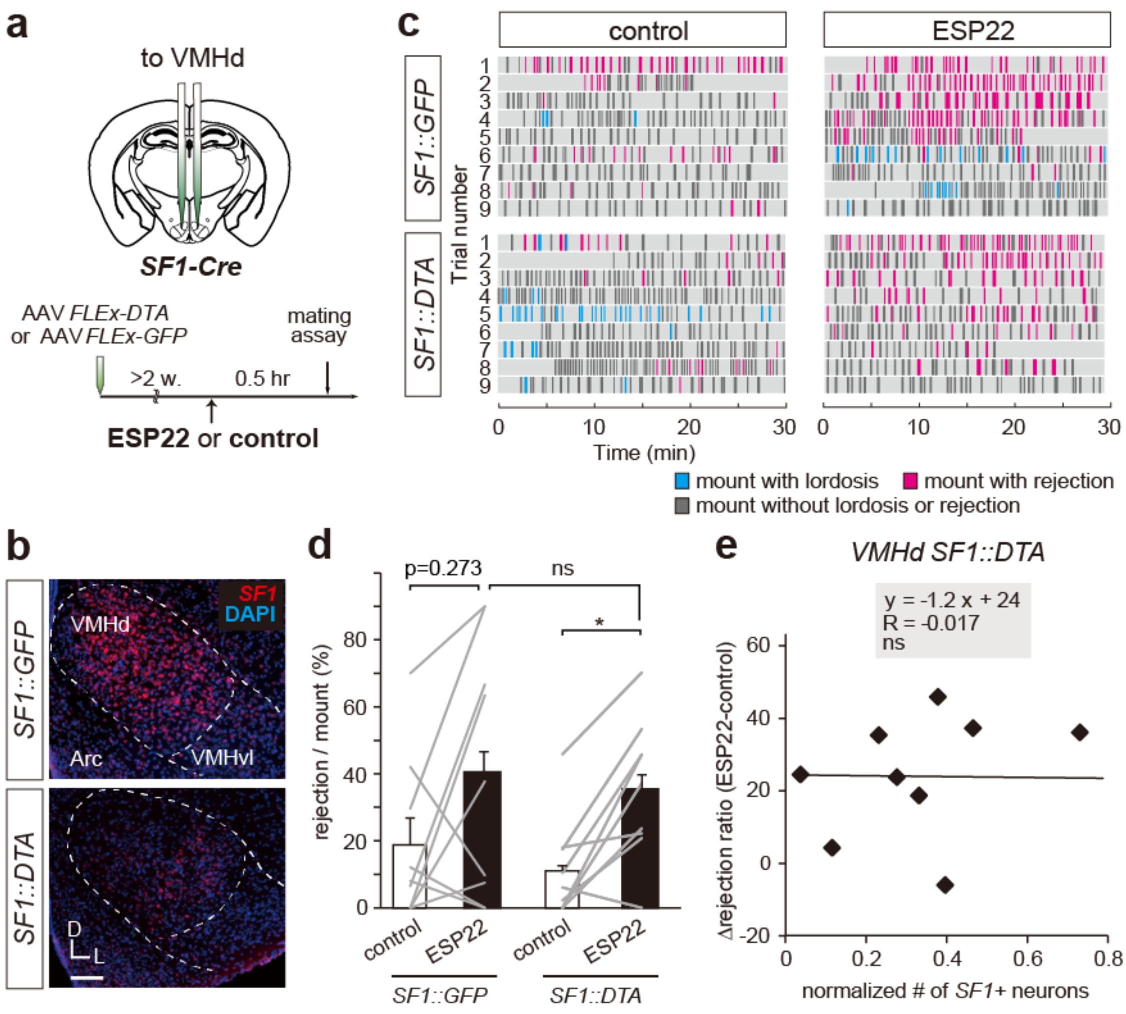

Supplementary Fig. 5 Genetic ablation experiments of *SF1*-positive neurons in the VMH. **a** Schematic illustration of the setup and timeline of genetic ablation experiments targeting the *SF1*-positive neurons in the VMHd. Image adapted from the Allen Mouse Brain Atlas (©2004 Allen Institute for Brain Science. Allen Mouse Brain Atlas. Available from: mouse.brain-map.org). **b** Representative ISH images of *SF1* expression (red) in VMH coronal sections from *SF1-Cre* female mice. D, dorsal and L, lateral. Scale bar, 100 μm. **c** Raster plots as detailed in **Fig. 1b**. n = 9. **d** Quantification of the rejection ratio of female mice pre-exposed to control buffer or ESP22. Error bars, S.E.M. n = 9. ns, not significant. *$p<0.05$ by Wilcoxon signed rank test with Bonferroni correction. **e** Correlation between the degree of loss-of-function (defined by the number of remaining *SF1*-positive neurons; *x*-axis) and the degree of ESP22-mediated sexual suppression (Δrejection ratio, the rejection ratio of the control buffer-exposed trial subtracted from the ESP22-exposed trial; *y*-axis). Rhombuses represent individual data for the loss-of-function experiments. Black line shows linear regression fitting.

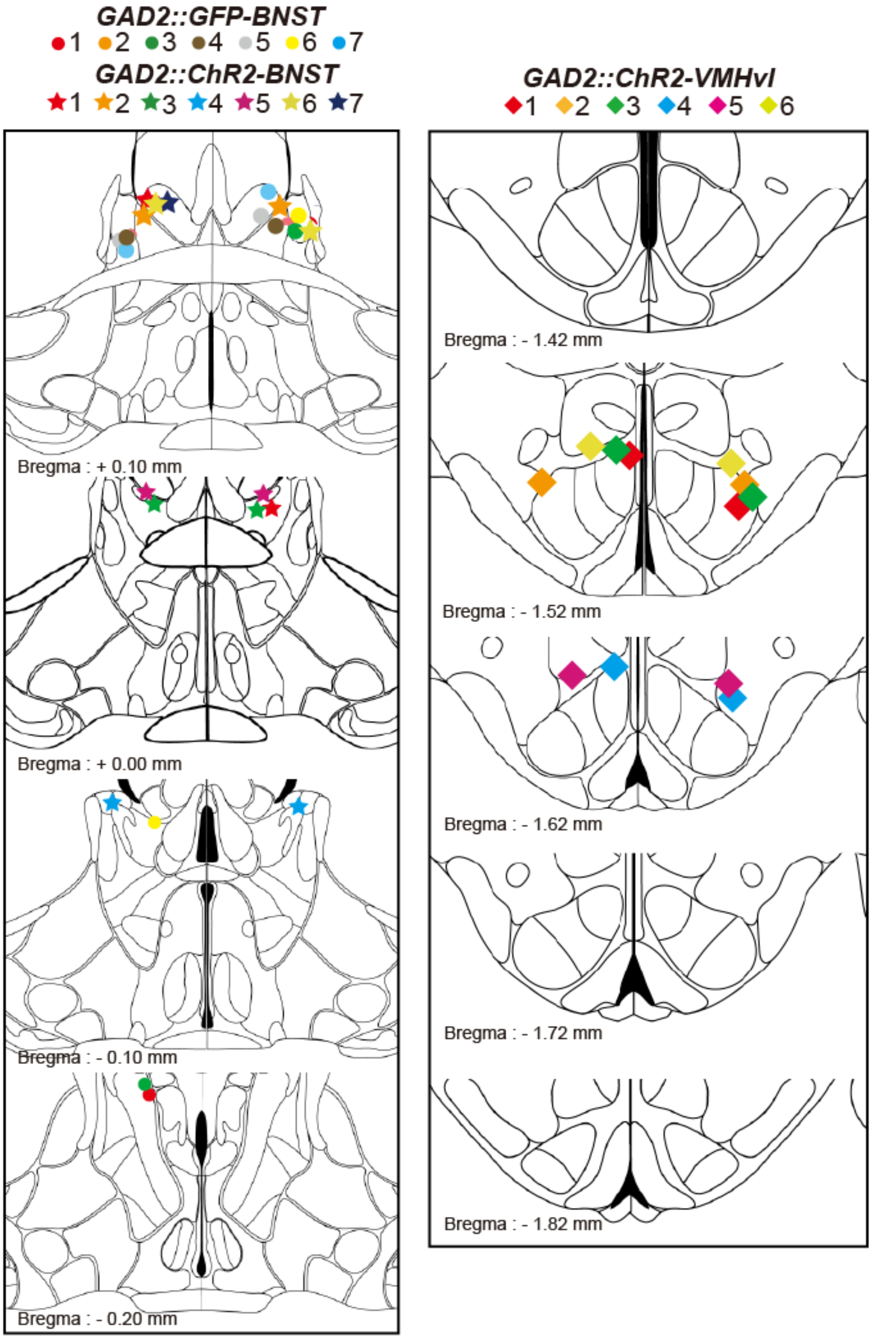

Supplementary Fig. 6 Histological verification of fiber optic locations in the BNST and VMHvl. Schematic image showing the location of fiber tips from all animals. Brain atlas was reproduced from Allen Mouse Brain Atlas (©2004 Allen Institute for Brain Science. Allen Mouse Brain Atlas. Available from: mouse.brain-map.org) with the location along the anterior (A)- posterior (P) axis shown on the left side at a distance of 1 mm from Bregma.

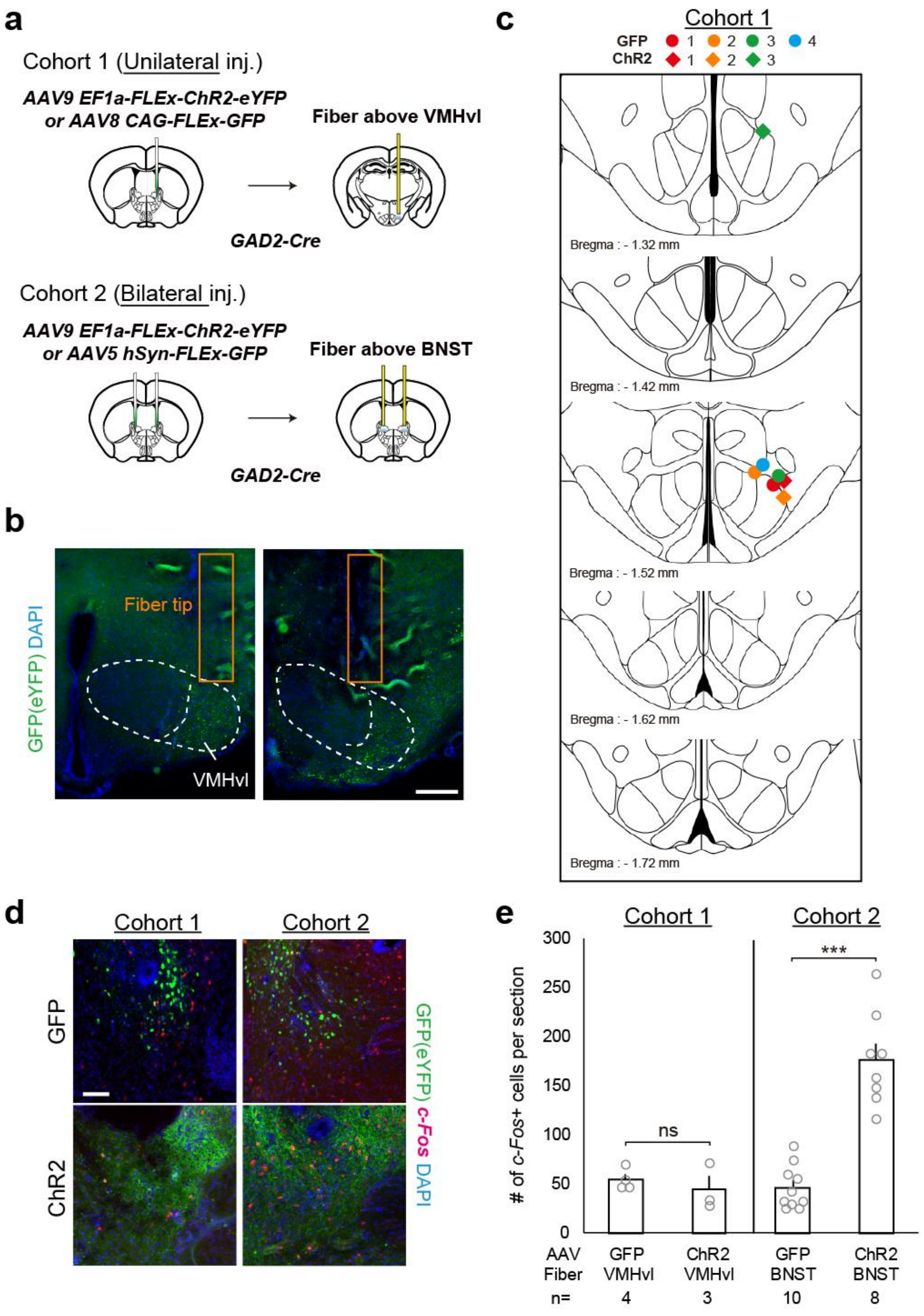

Supplementary Fig. 7 Optogenetic activation of axons from *GAD2*-positive BNST neurons in the VMHvl does not activate cell bodies in the BNST by back-propagating action potentials.

a Schematic of experiment design. Cohort 1: *GAD2-Cre* male mice received unilateral injection of Cre-dependent AAV to express into BNST either ChR2 or GFP as control. An optic-fiber was placed above VMHvl. Cohort2: *GAD2-Cre* male mice received bilateral injection of Cre-

dependent AAV to express either ChR2 or GFP as control. Optic-fibers were placed above BNST. Image adapted from the Allen Mouse Brain Atlas (©2004 Allen Institute for Brain Science. Allen Mouse Brain Atlas. Available from: mouse.brain-map.org). **b, c** Location of fiber tips in cohort 1 animals. Brain atlas is reproduced from Allen Mouse Brain Atlas (©2004 Allen Institute for Brain Science. Allen Mouse Brain Atlas. Available from: mouse.brain-map.org). Scale bar, 200 µm. **d** Representative coronal sections showing ChR2-eYFP or GFP (green), and *c-Fos* mRNA expression (red) in the BNST. Scale bar, 100 µm. **e** Quantitative analysis of the number of *c-Fos*+ cells per section. The injected AAV, location of optic-fiber, and number of unilateral brain areas counted are noted within the figure. Error bars, S.E.M. n = 3–10. ns, not significant. ***p<0.001 by Wilcoxon rank sum test.
