# Supplementary material for: Sexual rejection via a vomeronasal receptor-triggered limbic circuit
Source: Nat Commun. 2018 Oct 26;9:4463. doi: 10.1038/s41467-018-07003-5 (PMC6203846; doi:10.1038/s41467-018-07003-5)
Supplement: Supplementary file 3 — Description of Additional Supplementary Files [file 41467_2018_7003_MOESM3_ESM.pdf]

Description of Additional Supplementary Files

File Name: Supplementary Movie 1

Description: 1 A C57BL/6J adult female mouse displays ESP22-mediated sexual rejections (4.33 MB). Video showing a social interaction between a C57BL/6J adult female mouse and a stud ICR male mouse. An ESP22-stimulated female mouse displays rejecting postures upon mounting.
